# Efficacy of cannabinoids compared to the current standard treatments on symptom relief in persons with multiple sclerosis (CANSEP trial): study protocol for a randomized clinical trial

**DOI:** 10.3389/fneur.2024.1440678

**Published:** 2024-07-24

**Authors:** Amel Zertal, Kanza Alami Marrouni, Nathalie Arbour, Didier Jutras-Aswad, Marie-Pascale Pomey, Isabelle Rouleau, Alexandre Prat, Catherine Larochelle, Pierre Beaulieu, Laury Chamelian, Marie-Pierre Sylvestre, Danielle Morin, Jean-Sylvain Ouellette, Nathalie Fréjeau, Pierre Duquette

**Affiliations:** ^1^Centre de Recherche du Centre Hospitalier de l'Université de Montréal (CRCHUM), Montreal, QC, Canada; ^2^Département de Neurosciences, Faculté de Médecine, Université de Montréal, Montreal, QC, Canada; ^3^Département de Psychiatrie et d’addictologie, Faculté de Médecine, Université de Montréal, Montreal, QC, Canada; ^4^Centre Hospitalier de l’Université de Montréal (CHUM), Montreal, QC, Canada; ^5^Département de Gestion, d’évaluation et de politique de santé, École de santé publique, Université de Montréal, Montreal, QC, Canada; ^6^Centre d’Excellence sur le Partenariat avec les Patients et le Public, Université de Montréal, Montreal, QC, Canada; ^7^Département de Psychologie, Faculté des Sciences Humaines, Université du Québec à Montréal, Montreal, QC, Canada; ^8^Département d'anesthésiologie et de Médecine de la Douleur, Faculté de Médecine, Université de Montréal, Montreal, QC, Canada; ^9^Département de Pharmacologie et Physiologie, Faculté de Médecine, Université de Montréal, Montreal, QC, Canada; ^10^Département de Médecine Sociale et Préventive, École de Santé Publique, Université de Montréal, Montreal, QC, Canada; ^11^MS Canada, Toronto, ON, Canada

**Keywords:** multiple sclerosis, spasticity, cannabinoids, tetrahydrocannabinol, cannabidiol, complementary treatment, randomized controlled trial

## Abstract

**Background:**

Multiple sclerosis (MS) is an inflammatory and degenerative disease of the central nervous system. More than 90,000 Canadians are affected; a cure is yet to be found. Available treatments to manage the disease course are only partially effective. For many years, persons with MS (PwMS) have used cannabis to relax, to reduce pain and spasticity, or to improve sleep and daily functioning, despite the lack of scientific evidence on the efficacy of specific cannabinoids [i.e., tetrahydrocannabinol (THC) and cannabidiol (CBD)] on these MS symptoms. The purpose of this clinical trial is to assess the effectiveness of different doses of these cannabinoids, alone or combined, on spasticity relief, compared to placebo. Moreover, we aim to determine which treatment is best effective to address other key MS conditions.

**Methods:**

A double-blinded, randomized, factorial, placebo-controlled trial will be performed. We intend to include up to 250 PwMS aged over 21 recruited from the Centre hospitalier de l’Université de Montréal MS Clinic. PwMS will be randomly assigned on a 1:1:1:1 ratio to one of the trial arms: THC alone, CBD alone, THC/CBD combination, or placebo, using stratified blocked randomization, with random blocks within each stratum. The primary outcome is a self-assessment of spasticity using the mean Numeric Rating Scale score over 7 days. The main outcome will be the difference in this score at 4 weeks compared to baseline. Secondary outcomes include assessments of spasticity as measured by a clinician, pain, fatigue, sleep, bowel, bladder, and sexual dysfunction, restless legs syndrome, mental health, quality of life, mobility, cognitive functioning, and adverse events. Treatment responders are eligible for a 12-week extension phase, using the same treatment allocation and assessments.

**Discussion:**

Previous clinical studies examined the efficacy of cannabis-based medicines in PwMS, mostly using products with 1:1 THC/CBD ratio. The major barrier to effectively use cannabis in real-world clinical settings is the lack of evidence on benefits of specific cannabinoids and information on possible related risks. The CANSEP study will contribute to overcome these limitations and identify the risks and benefits of cannabis-based treatments in PwMS.

**Clinical trial registration:**

ClinicalTrials.Gov, NCT05092191.

## 1 Introduction

Multiple sclerosis (MS) is an inflammatory disease of the brain and spinal cord afflicting over 90,000 Canadians (1). MS causes numerous symptoms, such as spasticity, pain, sphincter and sleep dysfunction, fatigue and depression (2). Spasticity has been reported in up to 80% of persons with MS (PwMS) (3). It is described as an involuntary increase in muscle tone, tightness, and spasms of the affected limbs. It is part of the upper motor neuron syndrome, which also comprises weakness, increased deep tendon reflexes, and the Babinski sign (4, 5).

Despite the growing number of available disease-modifying treatments, none are curative (6, 7). PwMS still carry a heavy burden of undermanaged symptoms (8). Current spasticity therapeutic approaches include muscle relaxants such as baclofen, tizanidine, and clonazepam. Injections of botulinum toxin are used for focal spasticity (9, 10). They are effective but must be repeated every 3 to 4 months. Diffuse and extreme spasticity responds well to intrathecal injections of baclofen delivered through an intra-abdominal programmed pump reservoir (11).

Cannabinoids have also been proposed as a potentially useful addition to current therapies to treat MS symptoms. Studies conducted in the United States, where cannabis is legal in most states, have reported that 35 to 40% of PwMS use cannabis (12, 13). Both prescribed and non-therapeutic cannabis products are legally accessible since 2018 to all adults in Canada (14). Sixty-five percent of Canadian PwMS have already been using cannabis to ameliorate mood, improve sleep, and/or relieve pain and spasticity (15). Analgesic, antihyperalgesic, neuro-protective, and anti-inflammatory properties have been attributed to tetrahydrocannabinol (THC) and cannabidiol (CBD) (16). A systematic review concluded that oromucosal nabiximols (~1:1 THC:CBD ratio) are safe and efficient to treat MS spasticity (17–19).

Unfortunately, whether specific cannabis derivatives (CBD vs. THC) or other forms can be specifically optimized to manage MS symptoms remains largely unexplored. A variety of cannabinoid-based products are now available and widely used by PwMS, as mentioned previously, despite the lack of robust scientific evidence to guide their decision. Few studies have systematically compared the two main cannabinoids (THC and CBD) individually or combined for the treatment of spasticity (20), but no Canadian trial has previously compared the cannabinoids. Furthermore, the potential mechanisms mediating the therapeutic and/or adverse events (AEs) (e.g., gastrointestinal and central nervous system such as psychopathologic/cognitive AEs) of cannabis-based medicines in PwMS are poorly documented (20).

In response to this gap in knowledge, this trial will document medical cannabis (THC, CBD, and their combination) as a novel treatment with the potential to improve spasticity and other MS symptoms and produce evidence-based knowledge to guide its use.

### 1.1 Hypothesis

We hypothesize that the administration of different doses of THC alone, CBD alone, and THC and CBD combined will result in a significant relief of spasticity compared to placebo.

### 1.2 Study objectives

The main aims of this study are: (1) to compare the efficacy of THC and CBD, alone and in combination, as add-on therapies to the current standard treatments for relief of spasticity in PwMS, (2) to assess the tolerability profile of THC and CBD, alone and in combination, (3) to identify the mechanisms underlying such therapeutic and adverse effects, considering sex, age, pharmacology, and immune profile.

## 2 Methods/design

### 2.1 Study overview

CANSEP is the first Canadian randomized clinical trial focusing on interventions with THC, CBD, or their combination, for the treatment of spasticity in PwMS. Our study is part of the Integrated Cannabis Research Strategy funded by the Canadian Institutes of Health Research (21), and MS Canada. The overarching vision of the Integrated Cannabis Research Strategy is to catalyze future research related to the health impacts of cannabis legalization, to validate the potential therapeutic benefits of cannabis, to understand risks and harms, and to support policy and regulatory models for studying cannabis use. Thus, CANSEP will explore in depth the potential therapeutic benefits and harms associated with cannabis use to strengthen the evidence base and to build cannabis-related research capacity in the field of MS. The study duration is 5 years as summarized in the schedule presented in the Supplementary material. Recruitment has begun on November 10, 2022. As of May 29, 2024, 50 participants have been enrolled, and of these, 41 have been randomized.

### 2.2 Study design

**Figure 1:**
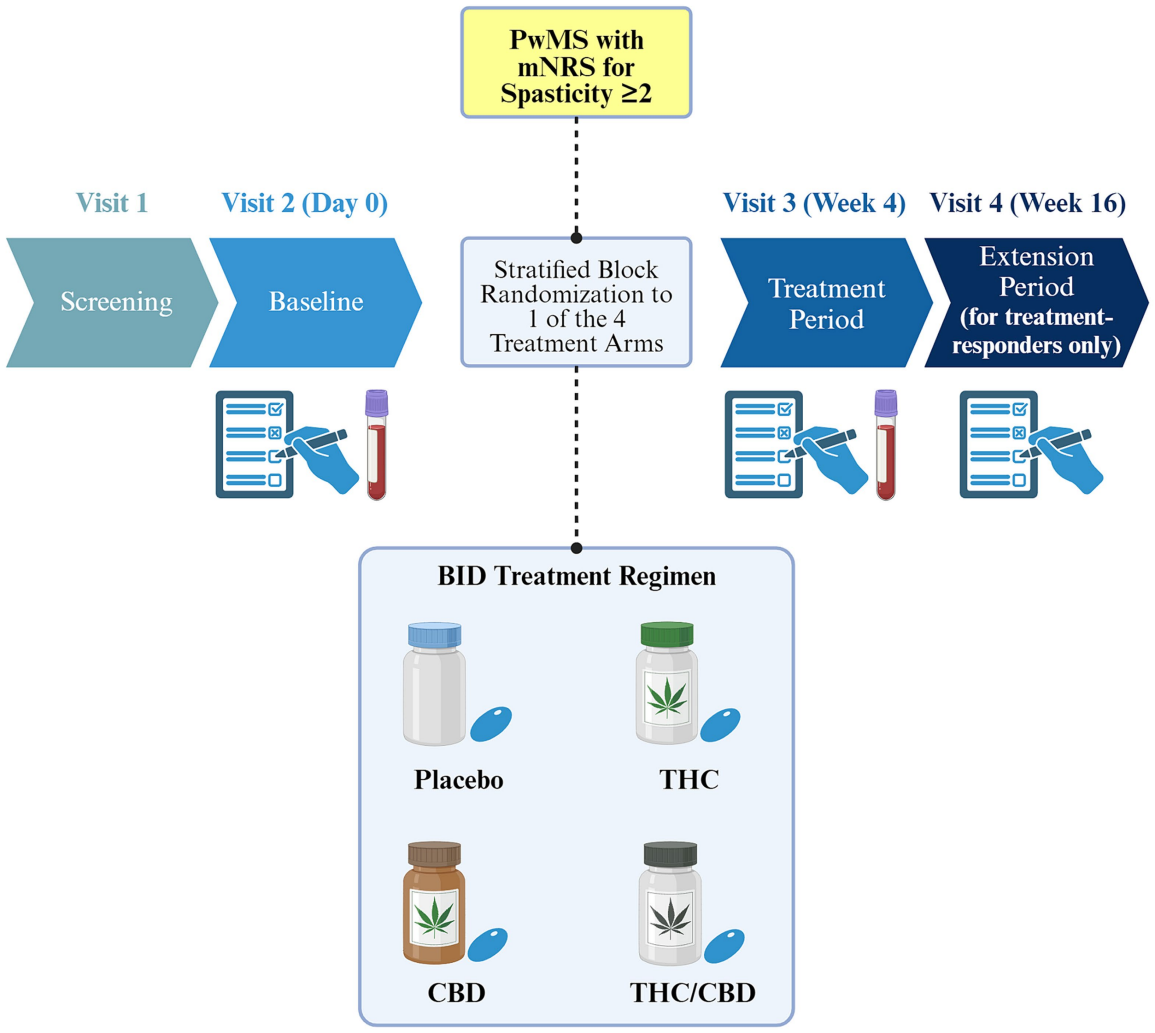
Overview of the CANSEP study design. Only treatment responders are eligible to the extension phase (i.e., those with an improvement of at least one point on the mNRS). Blood samples will be collected at baseline and after the treatment period, while primary, secondary, and exploratory outcomes will be assessed starting from the baseline visit. Different treatment bottles are used for illustrative purposes only. PwMS, persons with multiple sclerosis; mNRS, mean score of the Numeric Rating Scale over 7 days; CBD, cannabidiol; THC, tetrahydrocannabinol; BID, twice a day. Created with BioRender.com.

CANSEP is a double-blinded, randomized, factorial, placebo-controlled trial in a cohort of PwMS with an incomplete response to standard treatments, using various measurements, and thorough, multidisciplinary outcomes. Up to 4 visits are scheduled during this trial. Visit 1 is dedicated to the consent process and the confirmation of eligibility criteria. Outcomes assessments are done during visit 2 (baseline) and during visit 3 which takes place after 4 weeks of treatment. Finally, outcomes assessments are also carried out during a fourth visit which is scheduled 12 weeks after the end of the initial treatment period for responders only, i.e., patients who have a decrease in mean Numeric Rating Scale (NRS) of at least one point compared to baseline. Indeed, spasticity will be assessed using the NRS (22), but the spasticity outcome analyzed in this trial will be the mean NRS score over 7 days (mNRS). Blood samples will be collected to measure immunological and neurobiological markers in order to investigate the underlying physiological responses of each treatment administration, and to identify potential mechanisms of their therapeutic effects. The study plan is represented in Figure 1.

### 2.3 Study population

The CANSEP study seeks to identify a cohort of PwMS according to the 2017 revised McDonald criteria (23), from the Centre hospitalier de l’Université de Montréal (CHUM) MS Clinic. Our study sample will be representative of the sex distribution of MS, which is 2.6 higher in women than in men in Canada (1). It will recruit adults of at least 21 years of age [the legal age in the province of Quebec to use cannabis products (24)], who reported a mean level of spasticity over 7 days of 2 points or more on the mNRS. All potential participants will be screened to determine their eligibility according to the following inclusion and exclusion criteria:

- Inclusion criteria: (a) Be diagnosed with MS (any subtype), for at least six months, by a MS neurologist, according to the 2017 revised McDonald criteria (23); (b) Spasticity or symptoms related to spasticity due to MS of at least one-month duration and not relieved with current therapy, at a mean level of 2 or more on the mNRS; (c) Have a stable dose of standard therapies for at least 4 weeks prior to the screening visit and willingness to maintain such therapies for the duration of the study; (d) Aged 21 years or older (24); (e) Have the ability, in the investigator’s opinion, and willingness to comply with all study requirements; (f) Able to speak and read French or English (grade nine level of language required).
- Exclusion criteria: (a) Concomitant disease with symptoms of spasticity, or that may have influenced their level of spasticity; (b) Received a botulinum toxin injection within four months prior to the screening visit or unwillingness to stop receiving botulinum toxin injections for the duration of the study; (c) Use of cannabis or cannabinoid-based products within 7 days prior to study entry and unwillingness to abstain from use of cannabinoids for the duration of the study; (d) History of schizophrenia, other psychotic illness, or other significant psychiatric disorder other than anxiety or depression associated with their underlying condition; (e) Alcohol or substance abuse disorder other than nicotine; (f) History of epilepsy or recurrent seizures; (g) Hypersensitivity to cannabinoids or any of the excipients of the study medication; (h) Clinically relevant cardiac dysfunction within the last 12 months or had a cardiac disorder that, in the opinion of the investigator, would put the subject at risk of a clinically relevant arrhythmia or myocardial infarction; (i) Impaired renal function, i.e., serum creatinine clearance lower than 50 mL/min; (j) Significantly impaired hepatic function, at visit 1, in the investigator’s opinion and/or had liver function tests of equal to or greater than three times the upper limit of normal; (k) Pregnancy or breastfeeding; (l) Men with history of fertility problems and who plan to conceive at any time in the future; (m) Any participant who plans to conceive either at screening or while enrolled in the study; (n) Inability (or unwillingness) of women of childbearing potential and men to use a medically acceptable form of contraception throughout the study duration; (o) Any other significant disease or disorder which, in the opinion of the investigator, may either put the subject at risk because of participation in the study, may influence the result of the study, or the subject’s ability to participate in the study; (p) Intention to travel internationally, or to donate blood during the study.

### 2.4 Randomization

Patients who meet all the inclusion criteria and none of the exclusion criteria and consent to take part in the trial after they have received the study oral and written information, will then be randomly assigned in equal proportions to either THC, CBD, THC + CBD, or placebo using stratified block randomization. Randomization will be stratified by sex and baseline mean spasticity score. In each of the 4 strata, corresponding to different combinations of sex (male vs. female) and baseline mean spasticity score ([2-6[vs. ≥6), randomization will be performed using blocks of size 4. This will minimize the imbalance between group sizes, while preventing unblinding of the treatment allocation of consecutive participants. The randomization schedule will be generated by CHUM’s Centre for the Integration and Analysis of Medical Data (CITADEL), who will send the randomization codes to the CHUM research pharmacy and keep secure digitalized copies. Randomization codes will be maintained throughout the study.

### 2.5 Intervention

#### 2.5.1 Arms and intervention

An authorization to conduct the CANSEP trial using THC and CBD manufactured by PurCann Pharma was obtained from Health Canada prior to the start of recruitment.

Participants will be allocated to four arms and initially receive THC (4 mg/day), CBD (40 mg/day), THC/CBD combination (THC 4 mg/day and CBD 40 mg/day), or placebo, on the first day. Every two days, the daily quantity will be multiplied by two up to a maximum of 20 mg for THC and 200 mg for CBD, if well tolerated (Table 1). The selection of doses is based on previous studies on cannabinoids in MS (19, 25–34), and on a systematic review indicating that a daily dose of 200 mg of CBD is effective for other neurological conditions and symptoms while being well tolerated (35). One of the authors’ expert opinion on cannabinoids in psychiatric research and clinical practice (36, 37), and the approved and available orally formulated cannabinoids in Canada (38, 39) also accounted for these doses’ selection. THC and CBD will be taken as softgels (cannabis extract; placebo will taste and look exactly the same), in two divided doses per day at 12-h intervals. Participants will receive the allocated treatment for a total of 4 consecutive weeks, followed by an additional 12 weeks of treatment for responders who will be identified as those who have a decrease from baseline in spasticity of at least one point on the mNRS. Participants enrolled in the trial will continue stable doses of other standard treatments for spasticity including muscle relaxants (baclofen, tizanidine, and clonazepam) (18).

**Table 1:** Medication dosage.

Treatment period (4 weeks)					
Week	Day	Daily THC dose (mg)^a^	Daily CBD dose (mg)^a^	Daily THC and CBD combined dose (mg)^a^	
				THC	CBD
W1	#1	4	40	4	40
	#2	4	40	4	40
	#3	8	80	8	80
	#4	8	80	8	80
	#5	16	160	16	160
	#6	16	160	16	160
	#7	20	200	20	200
W2 to W4 included		20	200	20	200
Extension period (12 weeks)					
Week		Daily THC dose (mg)^a^	Daily CBD dose (mg)^a^	Daily THC and CBD combined dose (mg)^a^	
				THC	CBD
W5 to W16 included		20	200	20	200

^a^Treatment will be taken in two divided doses per day at 12-h intervals. CBD, cannabidiol; THC, tetrahydrocannabinol; W, week.

#### 2.5.2 Medical management

Regardless of the assigned arm, participants will receive medical examination and management from the study physician according to usual standards of care. Medication adherence will be monitored using a daily diary. The study pharmacist will count the unused capsules at the end of the 4 initial weeks of treatment and during the additional period of 12 weeks and will check for accuracy by comparing with the numbers mentioned on the daily diary reported by the participants.

### 2.6 Assessments

#### 2.6.1 Screening

At the screening visit, participants will be asked to provide sociodemographic information, their medication use, and their comorbidities. Furthermore, compulsive use and the individual’s preoccupation with cannabis use will be assessed by the Severity Dependence Scale, a self-reported questionnaire (40). The Structured Clinical Interview for DSM-5 Disorders will be administered to assess substance use disorders, including alcohol and other substances (41). The heart condition will be reviewed by electrocardiography. A pregnancy test and birth control questionnaire will be conducted in women aged ≤50 years with no sign of menopause and who did not undergo surgical contraception. A MS neurologist will evaluate the participants’ eligibility and will assess spasticity and disability with the Modified Ashworth Scale and the Expanded Disability Status Scale, respectively (42, 43).

#### 2.6.2 Primary outcome

The primary outcome (i.e., patient-reported spasticity) will be assessed using the mNRS (22, 44). The main outcome will be the difference in mNRS recorded for 7 days prior to the visit at week 4 and the visit at baseline. NRS is the most used tool to assess spasticity in most previous RCTs of treatment, including cannabinoid-based therapy, for MS-related spasticity (22). It is represented on a 0–10 scale, where 0 means no spasticity and 10, the worst possible spasticity (22). The NRS has been shown to be more robust than the Ashworth Scale for the test–retest reliability and highly correlative of the Patient Global Impression of Change scores (22, 44). Most studies investigating cannabis-derived products enrolled MS patients who have NRS ≥4 on the 0–10 scale (22). To include patients with moderate spasticity, we also enrolled those who have mNRS ≥2 on the 0–10 scale (17, 22, 44).

#### 2.6.3 Secondary and exploratory outcomes

All the secondary – efficacy and safety – outcomes will be assessed at baseline, at week 4 and 12 weeks after the initial treatment period. Clinical efficacy outcomes are presented in Table 2. In addition, we will assess the success of participants’ blinding after the initial treatment period with the James Blinding Index (69). Blood tests will be conducted only at baseline and at week 4 to measure immunological and neurobiological markers. Safety measures will include all reported or observed adverse events and serious adverse events (AEs and SAEs). Pregnancy and birth control will be re-evaluated every 4 weeks.

**Table 2:** Secondary and exploratory efficacy assessments.

Test	Symptom	Type of outcome
Modified Ashworth Scale (42)	Spasticity, as assessed by a clinician	ClinRO
Expanded Disability Status Scale (43)	Disability	
Positive and Negative Syndrome Scale (45)	Psychotic symptoms	
Multiple Sclerosis Quality of Life Inventory (46, 47), including^a^:		PRO
MOS Pain Effects Scale (46, 47)	Pain	
Modified Fatigue Impact Scale – 5-Item Version (46, 47)	Fatigue	
Bowel Control Scale (46, 47)	Bowel dysfunction	
Bladder Control Scale (46, 47)	Bladder dysfunction	
Modified Social Support Survey – 5-Item Version (46, 47)	Perceived social support	
Sexual Satisfaction Scale (46–48)	Sexual dysfunction	
36-Item Short Form Survey (46, 47)	Quality of life	
Perceived Deficits Questionnaire (46, 47, 49)	Subjective cognitive function	
Pittsburgh Sleep Quality Index (50) and Epworth Sleepiness Scale (51)	Sleep issues, according to the assessment of sleep quality and sleepiness, respectively	
Restless Legs Syndrome Severity Rating Scale (52, 53)	Restless legs syndrome’s severity	
Hospital Anxiety and Depression Scale (54, 55)	Anxiety and depression	
Cannabis Experience Questionnaire (56, 57)^b^	Euphoric and paranoid-dysphoric effects of cannabis	
Battery of cognitive tests: Montreal Cognitive Assessment (58), Brief Visuospatial Memory Test–Revised (59), D-KEFS Color-Word Interference Test (60), Hopkins Verbal Learning Test–Revised (61), The Trial Making Test A/B (62), and Symbol Digit Modalities Test (63)	Objective cognitive function	PerfO
Timed 25 Foot-Walk test (64–66)	Mobility	

ClinRO, clinician-reported outcome; PRO, patient-reported outcome; PerfO, performance outcome. ^a^We adapted the Multiple Sclerosis Quality of Life Inventory for the CANSEP trial, by excluding two of its ten scales: (1) The Impact of Visual Impairment Scale was not included, because it is not widely used in clinical practice and not relevant to the CANSEP trial; (2) instead of the Mental Health Inventory, we considered the Hospital Anxiety and Depression Scale, a widely used instrument in clinical practice, but also a validated scale in the MS population (55, 67, 68). Furthermore, we added the Cannabis Experience Questionnaire for the subjective effects of cannabinoids (56, 57). ^b^All tests will be conducted at baseline, at week 4, and at week 16. However, participants who never used cannabis in their lifetime will not complete the Cannabis Experience Questionnaire at baseline.

### 2.7 Data analyses

The data will be analyzed once all randomized subjects have completed the trial. Analyses of primary and secondary outcomes will rely on the intention-to-treat paradigm. Thus, all initially randomized subjects will be included. Descriptive statistics will be used to compare the baseline characteristics of subjects. They will include means, medians, standard deviations and interquartile ranges for continuous variables, and frequencies and percentages for categorical variables. Sensitivity analysis will be performed to the first few participants who did not complete the NRS scale as per the last version of the protocol and consequently have a different primary outcome measure.

#### 2.7.1 Sample size and power calculation

The sample size for this trial was calculated using ‘pwr.t.test’ (for a Student t-test) in the ‘pwr’ package in R statistical software (70). The required sample size was estimated to ensure at least 80% power, with a two-sided alpha significance level of 5%, to detect clinically important effects of the two factors THC and CBD, on spasticity, which is the primary outcome (71). A target total recruitment of 200 patients would cover scenarios with a mean change from baseline among treated patients ranging from −1.90 to 1.55, where −1.55 is what we consider to be the minimal clinically significant effect (22, 33, 72, 73). However, some power would be lost upon Bonferroni adjustment. Thus, a target total recruitment of 250 patients would be more adequate, after accounting for a potential rate of attrition of 5% over the study period, based on previous studies (22, 33, 72, 73) and the clinical experience at the CHUM MS Clinic.

#### 2.7.2 Primary endpoints

Firstly, an analysis of variance (ANOVA) will be used to assess the treatments effect on the differences in the mNRS from baseline to week 4. The normality of the responses will be assessed using normal-quantile plots. The ANOVA model will only include the factors THC and CBD. Secondly, an analysis of covariance (ANCOVA) will be used to evaluate the treatments effect on the post-intervention mNRS while adjusting for baseline mNRS and sex (stratification variables). The ANCOVA may reduce potential bias present in the ANOVA model (74). Finally, the ANCOVA model described above will be expanded by including potential confounders, such as biomarkers, for which a clinically meaningful difference between any two groups will be revealed by the preliminary descriptive analysis. The estimated adjusted and unadjusted differences in mNRS from baseline to week 4 and their 95% confidence intervals will be reported for all models.

#### 2.7.3 Secondary and exploratory endpoints

The secondary and exploratory outcomes will be analyzed using the same methods as spasticity. Exploratory outcomes and mechanistic factors (sex, neurobiological markers, age, etc.) will be analyzed using ANCOVA models.

##### 2.7.3.1 Sex and gender-based analysis

Numerous publications have documented the impact of sex on multiple aspects of MS (biology, epidemiology, pregnancy) and substance use, respectively (75–78). Our statistical plan will include stratified analyzes to identify the sex-linked immune mechanisms as well as the sex-specific effects of cannabis.

##### 2.7.3.2 Safety analyses

AEs will be analyzed using the Common Terminology Criteria for Adverse Events (version 5.0) according to the Medical Dictionary for regulatory activities (79). The frequency and percentage of participants experiencing each specific AE will be tabulated by severity and treatment. Each AE will be counted once under the maximum severity or the strongest recorded causal relationship to the studied product. For each arm, all AEs will be grouped by organ class and, for each AE, the relative risk and absolute risks between arms with their 95% confidence intervals will be calculated.

#### 2.7.4 Oversight and monitoring

An independent data and safety monitoring board will review the accumulated data to assure that the safety of study participants is protected while the scientific goals of the study are being met. The data and safety monitoring board is responsible for conducting reviews of accumulating safety and efficacy data once a year. It may recommend halting or modifying study procedures if there is a clear and evident reason related to the safety of the study participants including and excess in frequency of any AE (judged by the data and safety monitoring board to be harmful to the participants) in one of the arms, and an excess in frequency of any SAE (grade 3 and higher) in one of the arms.

#### 2.7.5 Missing data and dropouts

Missing data could occur due to two types of reasons: a missing or illegible item response and a missed visit. We will report the percentage of missing values for each variable of interest by visit. Missing values for the primary outcome at follow-up will be imputed by the baseline value resulting in null change from baseline. No imputation for the primary outcome will be needed from the per-protocol analysis. For the ANCOVA models, imputation of missing covariate values will be handled by multiple imputation methods using chained equations. Since we cannot verify the missing at random hypothesis, which is required for the valid use of this imputation method, we will perform a sensitivity analysis with complete cases only.

#### 2.7.6 Sensitivity analysis

To test if our conclusions are robust to the invalidity of the hypothesis of no interaction between the factors, we will use an ANOVA model including THC, CBD, and their interaction. We expect to find a negligible interaction effect.

## 3 Discussion

Current therapeutic approaches for MS have partial efficacy. Disease-modifying therapies are limited in their action on MS symptoms (11). Both preclinical and clinical data support that analgesic, antihyperalgesic, neuroprotective, and anti-inflammatory properties have been attributed to cannabis-derived cannabinoids, essentially THC and/or CBD (30, 80). The CANSEP trial is part of a large Canadian Institutes of Health Research program on cannabis research in priority areas (21). It was developed in response to the lack of evidence about the safety and the efficacy of specific cannabinoids in MS (20). The originality of our study comes from the fact that we will compare the distinct and combined effects of THC and CBD on spasticity and other symptoms such as pain, sleep, well-being, and quality of life of Canadian PwMS.

CANSEP will fill a knowledge gap since no Canadian study has yet systematically compared the two main cannabinoids, THC and CBD, and their combination, to placebo, as an add-on treatment for spasticity. This will answer the urgent need to assess cannabinoids’ efficacy on spasticity and other symptoms in MS in addition to their acceptability and safety when used as an add-on therapy to usual standard treatments. Additionally, CANSEP can provide explanations for potential therapeutic mechanisms and/or AEs such as gastrointestinal and psychiatric/cognitive effects associated with each type of cannabis-based medication in PwMS that are still poorly understood (18). For example, the polymorphism in cannabinoid receptor 2 associated with reduced endocannabinoid effects is more prevalent in autoimmune diseases, including MS (81). Thus, CANSEP will provide a better characterization of CBD and THC influence on immune and pharmacological factors which could directly influence spasticity to guide both health professionals and PwMS in the decision-making process. Indeed, while our main concern is spasticity, we are also evaluating other MS symptoms (pain, fatigue, bladder, bowel, and sexual dysfunctions, mobility, the restless legs syndrome, mental health, cognition, and quality of life). The CANSEP trial’s results will impact how these different symptoms could be managed and will provide a better grasp of tolerance and toxicity. We have a long experience with MS patients, and the trial’s results will provide guidance to a better control of symptomatic aspects of MS with cannabinoids. It will also guide health professionals and PwMS on the treatment’s dose and frequency.

Another strength of this trial is the development of two ancillary studies. Participants are free to take part or not in these ancillary studies without affecting their participation in the CANSEP clinical trial.

- Ancillary study 1 – Perception of Canadian patients with MS on the use of cannabis for a better management of the disease symptoms (PerSPective Study): The main objective is to conduct a pan-Canadian online survey, co-created for and with PwMS to identify facilitating or limiting factors on the use of medical cannabis by PwMS to anticipate the strategies to be put in place depending on the results of the research.
- Ancillary study 2 – Patient experience assessment of participants in the CANSEP trial and the contribution of patient partner researchers in the implementation work of this project (EXPECT Study): This study includes quantitative questionnaires and semi-structured qualitative interviews to understand how the patient partners involved in CANSEP have been integrated and intervened in the realization of the project (80). A second objective is to assess the experience lived by the PwMS participating in the CANSEP trial and their perceptions related to the use of THC and CBD as a clinical intervention.

The limitations of the CANSEP trial may be related to data collection through patient-reported outcome measurements which could inaccurately estimate problems and not be suitable for all patients (82). However, a daily diary developed to monitor medication adherence and scheduled phone calls between visits will ensure proper data collection. Also, the variability in understanding the definition of spasticity among patients could be a limitation in the assessment of this symptom although we provide the same description to all participants.

## 4 Knowledge transfer

At the end of the study, we will conduct a robust knowledge transfer. We plan a variety of strategies (e.g., health cards and video clips) in collaboration with patient partners to train PwMS interested in using cannabinoids-based medicines in an appropriate level of literacy. For clinicians, we will create a tool of shared decision-making. To translate results to the health care level, we will build on our involvement in national/international research, clinical and training organizations. For the general population, we plan to organize public conferences to raise awareness of societal issues as well as participation in events organized yearly by the CHUM.

## 5 Conclusion

CANSEP will provide a better characterization of CBD and THC’s influence on spasticity and on immune and pharmacological factors which could directly influence spasticity treatments and guide both health professionals and PwMS in the decision-making care process.

## Ethics statement

The CANSEP study involving humans was approved by the Research Ethics Board of Centre de recherche du Centre hospitalier de l’Université de Montréal, on September 9, 2022 (approval #21.303). The study was conducted in accordance with the local legislation, institutional requirements and the Declaration of Helsinki. The participants provided their written informed consent to participate in this study.

## Author contributions

AZ: Methodology, Project administration, Supervision, Visualization, Writing – original draft, Writing – review & editing. KAM: Visualization, Writing – original draft, Writing – review & editing. NA: Conceptualization, Funding acquisition, Methodology, Writing – review & editing. DJ-A: Conceptualization, Funding acquisition, Methodology, Writing – review & editing. M-PP: Methodology, Writing – review & editing. IR: Methodology, Writing – review & editing. AP: Writing – review & editing. CL: Writing – review & editing. PB: Writing – review & editing. LC: Writing – review & editing. M-PS: Writing – review & editing. DM: Writing – review & editing. J-SO: Writing – review & editing. NF: Writing – review & editing. PD: Conceptualization, Funding acquisition, Methodology, Supervision, Writing – review & editing.
